# Development and characterization of carboxy-terminus specific monoclonal antibodies for understanding MUC16 cleavage in human ovarian cancer

**DOI:** 10.1371/journal.pone.0193907

**Published:** 2018-04-30

**Authors:** Abhijit Aithal, Wade M. Junker, Prakash Kshirsagar, Srustidhar Das, Sukhwinder Kaur, Catherine Orzechowski, Shailendra Kumar Gautam, Rahat Jahan, Yuri M. Sheinin, Imayavaramban Lakshmanan, Moorthy P. Ponnusamy, Surinder K. Batra, Maneesh Jain

**Affiliations:** 1 Department of Biochemistry and Molecular Biology, University of Nebraska Medical Center, Omaha, NE, United States of America; 2 Sanguine Diagnostics and Therapeutics Inc. Omaha, NE, United States of America; 3 Department of Pathology and Microbiology, University of Nebraska Medical Center, Omaha, NE, United States of America; 4 Fred and Pamela Buffett Cancer Center, University of Nebraska Medical Center, Omaha, NE, United States of America; Duke University School of Medicine, UNITED STATES

## Abstract

MUC16 is overexpressed in ovarian cancer and plays important roles in invasion and metastasis. Previously described monoclonal antibodies against cell surface expressed MUC16 recognize the N-terminal tandemly repeated epitopes present in cancer antigen 125 (CA125). MUC16 is cleaved at a specific location, thus, releasing CA125 into the extracellular space. Recent reports have indicated that the retained carboxy-terminal (CT) fragment of MUC16 might play an important role in tumorigenicity in diverse types of cancers. However, limited data is available on the fate and existence of CT fragment on the surface of the cancer cell. Herein, we characterize two monoclonal antibodies (mAbs) showing specificity to the retained juxtamembrane region of MUC16. For the first time, we demonstrate that MUC16 is cleaved in ovarian cancer cells (NIH:OVCAR-3 [OVCAR-3]) and that the cleaved MUC16 subunits remain associated with each other. Immunohistochemical analyses on different grades of ovarian tumor tissues indicated differential reactivity of CA125 and MUC16 CT mAbs. The CA125 (M11) mAb detected 32/40 (80%), while the CT mAb (5E6) detected 33/40 (82.5%) of total ovarian cancer cases. For serous and serous papillary cases, the CA125 (M11) mAb stained 27/31 cases (87%), while CT mAb (5E6) stained 29/31 cases (93.5%). The CT mAb(s) accurately predict expression of MUC16 since their epitopes are not tandemly repeated and their reactivity may not be dependent on O-linked glycosylation. These antibodies can serve as valuable reagents for understanding MUC16 cleavage and may also serve as potential therapeutic agents for treatment of ovarian cancer.

## Introduction

Cancer Antigen 125 (CA125) was first discovered in 1981 as a membrane antigen expressed by ovarian cancer cells [1]. Two independent reports later confirmed CA125 to be encoded by the MUC16 gene [2, 3]. MUC16 was subsequently identified as a high molecular weight, heavily glycosylated mucin involved in various physiological processes related to both normal and malignant conditions. MUC16 has a heavily O-glycosylated N-terminus and a tandem repeat region (60 tandem repeats of 156 amino acids each) that collectively comprises CA125; and a carboxy-terminal (CT) fragment. The CT fragment is interspersed with multiple sea urchin sperm protein, enterokinase and agrin (SEA) domains (that are potential cleavage sites), and contains a transmembrane domain that is followed by a 32 amino acid cytoplasmic tail with potential phosphorylation sites [4].

MUC16 is known to promote cancer invasion and metastasis [5–9] and inhibits host immune responses by directly down regulating the activity of NK cells [10, 11]. It has also been shown to selectively modulate drug response in ovarian and pancreatic cancer cells [5, 12]. It is believed (but not proven) that MUC16 undergoes cleavage in the penultimate SEA domain to generate circulating CA125 and a cell surface bound CT fragment [4, 13]. Recently, much interest has been garnered on the latter with multiple groups demonstrating its pro-tumorigenic and metastasis enhancing properties in both ovarian and pancreatic cancer [6, 8, 12, 14]. The mechanism of action seems to be dependent on AKT and ERK activation [6, 8, 15]. However, all these studies were carried out using transfected cells and to date limited information is available regarding the existence of an endogenous MUC16 CT fragment. The lack of antibodies with specificity for the retained CT has been central to this problem.

In this report, we present two characterized mAbs showing specificity to the retained CT fragment in order to understand MUC16 cleavage and its putative role in ovarian carcinogenesis. Our findings show that MUC16 is cleaved to generate an approximately 20kDa doublet of fragment(s) in OVCAR-3 cells and that the resulting subunits (CA125 and CT) are associated with each other. These mAbs give predominantly cytoplasmic staining in serous and serous papillary ovarian adenocarcinoma cases, with one of the mAbs giving equivalent sensitivity to that of CA125 antibody in human ovarian cancer tissues. Further, the unique location of the epitope allows the CT mAbs to bind the MUC16 CT at the cell surface, and hence can potentially be useful for targeting ovarian cancer. It is highly likely that binding of these reagents may be affected by CA125 cleavage, but may not be affected by serum CA125 levels.

## Materials and methods

### Animals

Female BALB/c mice were obtained from Harlan Sprague Dawley, Frederick, MD (acquired by Envigo Biosciences in 2015) and were housed in the Animal facility of the University of Nebraska Medical Center, Omaha, NE, USA. Mice were housed in standard cages dispensed with bedding, animal feed and Hydropac water pouches as per IACUC guidelines for housing mice in Animal Facility. All procedures were approved by the University of Nebraska Medical Centre Institutional Animal Care and Use Committee (IACUC) and in accordance to NIH guidelines (Approval Number: 12-042-07). Mice were monitored every alternate day for signs of distress and were euthanized humanely according to IACUC guidelines (100% CO2 inhalation followed by cervical dislocation). During the study no adverse episodes or signs of distress were reported and no anesthesia was administered at any time. No animal died during the experiment except when sacrificed at the experimental end point.

### Antibodies and cell lines

Anti-MUC16/CA125 mAb (M11) was procured from Dako (Agilent technologies, Santa Clara, CA). CA125 mAb (OC125 type) was purchased from Fitzgerald Industries International (North Acton, MA). (LUM16-4) rabbit antiserum [16] was a kind gift from Dr. Carlstedt, Lund University, Sweden. Monoclonal antibodies to the C-terminal 114 amino acid region of human MUC16 were generated as follows. The cDNA sequence encoding the C-terminal 114 amino acids of human MUC16 (Uniprot entry Q8WXI7) was cloned into the bacterial expression vector pET-28a and transformed into C41 (DE3) cells. After induction at 37°C with 1mM IPTG, bacterial extracts were made and the protein was purified by AKTA Ni-NTA affinity chromatography using standard methods [16]. Purified MUC16 CT was emulsified in Freund's Adjuvant, and used to immunize BALB/c mice. Hybridomas were made by fusion of spleen cells with P3X63Ag8.653 cells, and were screened using enzyme linked immunosorbent assay (ELISA) and flow cytometry as detailed below. Monoclonal antibodies from positive stable hybridomas were purified from enriched supernatants. P3X63Ag8.653, NIH:OVCAR-3 [OVCAR-3], SK-OV-3 [SKOV3] and MIA PaCa-2 cells were procured from ATCC (Manassas, VA, U.S.A.). OVCAR-5 cells were generously provided by Dr. John S. Davis (University of Nebraska Medical Center). OVCAR-5 and SKOV3 cell were cultured in DMEM supplemented with 10% fetal bovine serum (FBS). OVCAR-3 cells were cultured in advanced RPMI (GIBCO, Thermo Fisher Scientific, Waltham, MA, USA) supplemented with 10% FBS. P3X63Ag8.653 cells were grown in RPMI supplemented with 10% FBS.

### Enzyme linked immunosorbent assay

ELISA wells were coated overnight at 4°C with 1 μg/ml of MUC16 CT recombinant protein in PBS. After 2 washes in PBS, the plates were blocked by adding 200 μl of 10% skim milk in PBS for 2 hrs at 37°C. Plates were subsequently washed twice with PBS, followed by incubation with 100 μl of the hybridoma supernatant or test antibodies for 2 hrs at room temperature (RT), which was followed by three washes in PBS. Plates were further incubated with a 1:5000 dilution of horse radish peroxidase conjugated anti-mouse IgG for 60 min. Plates were washed three times and bound antibody was detected using TMB substrate.

### Flow cytometry

Cell surface flow cytometry was performed following a standard protocol as detailed previously [17]. Cells were incubated with anti-MUC16 antibodies for 60 min on ice followed by Alexa 488-conjugated goat anti-mouse IgG for 30–45 min on ice. Analysis was done on a BD LSRII or BD FACS CALIBUR flow cytometer. For some experiments, dead cells were excluded by staining with propidium iodide.

### Confocal microscopy

OVCAR-3 cells were seeded onto sterilized glass cover slips overnight. After washing with PBS twice, the cells were fixed with 4% paraformaldehyde in PBS (pH 7.4) for 10 min. After washing the cells twice with PBS containing 0.1M glycine, cells were permeabilized with 0.1% Triton X-100 in PBS. For non-permeabilization conditions, PBS alone was added. Blocking was done with 10% goat serum in PBS for 30 min, followed by incubation with the mAbs (10 μg/ml) diluted in PBS. Cells were washed 4 times with PBS containing 0.05% Tween 20 (PBST) and then incubated with Alexa Fluor 488 conjugated goat anti-mouse secondary antibodies for 45 min. Cells were washed 4 times with PBST, and mounted on glass slides in anti-fade Vectashield mounting medium containing DAPI (4′, 6-Diamidino-2-Phenylindole, Dihydrochloride) (Vector Laboratories, Burlingame, CA). Immunostaining was observed using a ZEISS LSM 710 microscope.

### Western blotting

Cells were washed twice in PBS and lysed in 1x RIPA buffer (50 mM Tris-HCl pH-7.5, 150 mM NaCl, 1% NP-40, 0.5% sodium deoxycholate, and 0.1% SDS) containing protease inhibitor mixture (Roche Diagnostics, Mannheim, Germany) and phosphatase inhibitors (1 mM NaF and 1 mM Na3VO4; Sigma Chemicals, St. Louis, MO) for 30 min on ice. Insoluble debris was removed by centrifugation (18000 g, 30 min, 4°C). Protein concentrations were determined using a BIO-RAD DC™ protein estimation kit (Bio-Rad Laboratories, Hercules, CA, USA). Lysates were resolved under reducing conditions by either by conventional SDS-PAGE (12%) [18] or by 0.1% SDS-2% agarose gel electrophoresis as described previously [19, 20]. The latter are useful for studying high molecular weight proteins that cannot enter standard SDS-PAGE gels and have been used before for studying other high molecular weight mucins [19–21]. Resolved proteins were transferred onto polyvinylidene difluoride (PVDF) membranes, blocked in 5% skim milk in 0.05% PBST, and probed with the respective antibodies. Anti-MUC16 CT mAb (3H1) was used at [0.5μg/ml]. Anti-MUC16 CT mAb (5E6) was used at [0.1μg/ml]. CA125 mAb (M11) was used at 1:1000. Anti-human β-actin (1∶10000, Sigma-Aldrich, St. Louis, MO, USA) was used as protein loading control. After washing extensively (3x10 min, PBST), membranes were probed with horseradish peroxidase-conjugated goat anti-mouse secondary antibody IgG (1:3000, Thermo Fisher Scientific, Waltham, MA, USA). Blots were washed (3x10 min, PBST) and processed with an ECL Chemiluminescence kit (Thermo Fisher Scientific, Waltham, MA, USA). The signal was detected by exposing the processed blots to X-ray film (Biomax Films, Kodak, NY).

### Immunoprecipitation

Cells were lysed in 1X PBS containing 0.5% Triton X-100, protease inhibitor mixture (Roche Diagnostics, Mannheim, Germany) and phosphatase inhibitors (1 mM NaF and 1 mM Na3VO4; Sigma Chemicals, St. Louis, MO). Insoluble debris was removed by centrifugation (18000 g, 30 min, 4°C). Cell lysates were pre-cleared for 1 hr at 4°C with 20μl of Protein A/G agarose beads, and incubated overnight at 4°C with 40μl of Protein A/G agarose beads pre-bound with 2μg of antibody or 1μl of neat antiserum. Immune complexes were washed 3 times with wash buffer (0.2% (v/v) Triton X-100 in PBS) and were eluted by boiling with 2X reducing/non-reducing SDS sample buffer. The immunoprecipitated proteins were resolved on 12% SDS-PAGE gels and western blotting was performed as described earlier.

### Transfection and expression of MUC16 CT fragments

HEK293T cells were transfected with constructs representing different lengths of the MUC16 CT [13] using Lipofectamine 2000 (Invitrogen, Carlsbad, CA, USA) according to the manufacturer’s instructions. Expression was checked using appropriate anti-FLAG, anti-HA, and MUC16 CT antibodies by western blot analysis of the extracted lysates.

### Immunohistochemistry

Ovarian cancer tissue microarrays (BIOMAX Cat# OV1004, Derwood, MD) were de-paraffinized in xylene, and rehydrated in a decreasing ethanol series. Endogenous peroxidase activity was quenched by incubating sections in 0.3% H_2_O_2_ in methanol for 15 min. Antigen retrieval was carried out by boiling the samples in Tris-EDTA buffer (10mM Tris, 1mM EDTA, 0.05% Tween 20, pH 9.0) for CT mAbs. For CA125 mAb, citrate buffer (10mM sodium citrate, 0.05% Tween 20, pH 6.0) was used. The tissue microarrays were blocked by incubating the sections with normal horse serum for 60 min at RT. Sections were then incubated with anti MUC16 antibodies (5E6 at 0.1μg/ml; CA125 at 1:1000) diluted in PBS overnight at 4°C. The slides were washed with PBST (3x5 min) followed by incubation with secondary antibody for 30 min. Slides were washed with PBST (3x5 min) and were incubated with DAB (Vector Laboratories, Burlingame, CA) until color development was evident. The slides were washed in water and counterstained with hematoxylin for 30 seconds. Slides were washed in water and dehydrated in an increasing ethanol series, followed by xylene and mounting with Permount permanent mounting media (Fisher Scientific, Fair Lawn, NJ). All stained slides were scored by a certified pathologist. Staining intensity was assessed using a scale of 0–3 (0-negative, 1-weak, 2-moderate, 3-strong immunoreactivity) and percentage of cells positive within a given case. The score of the staining intensity and the percentage of immunoreactive cells were then multiplied to obtain a H-score ranging from 0 to 3. A section was considered “positive” for MUC16 if the staining intensity was at least 1.

## Results

### Anti-MUC16 CT mAbs recognize a cell surface exposed epitope

**Fig 1:**
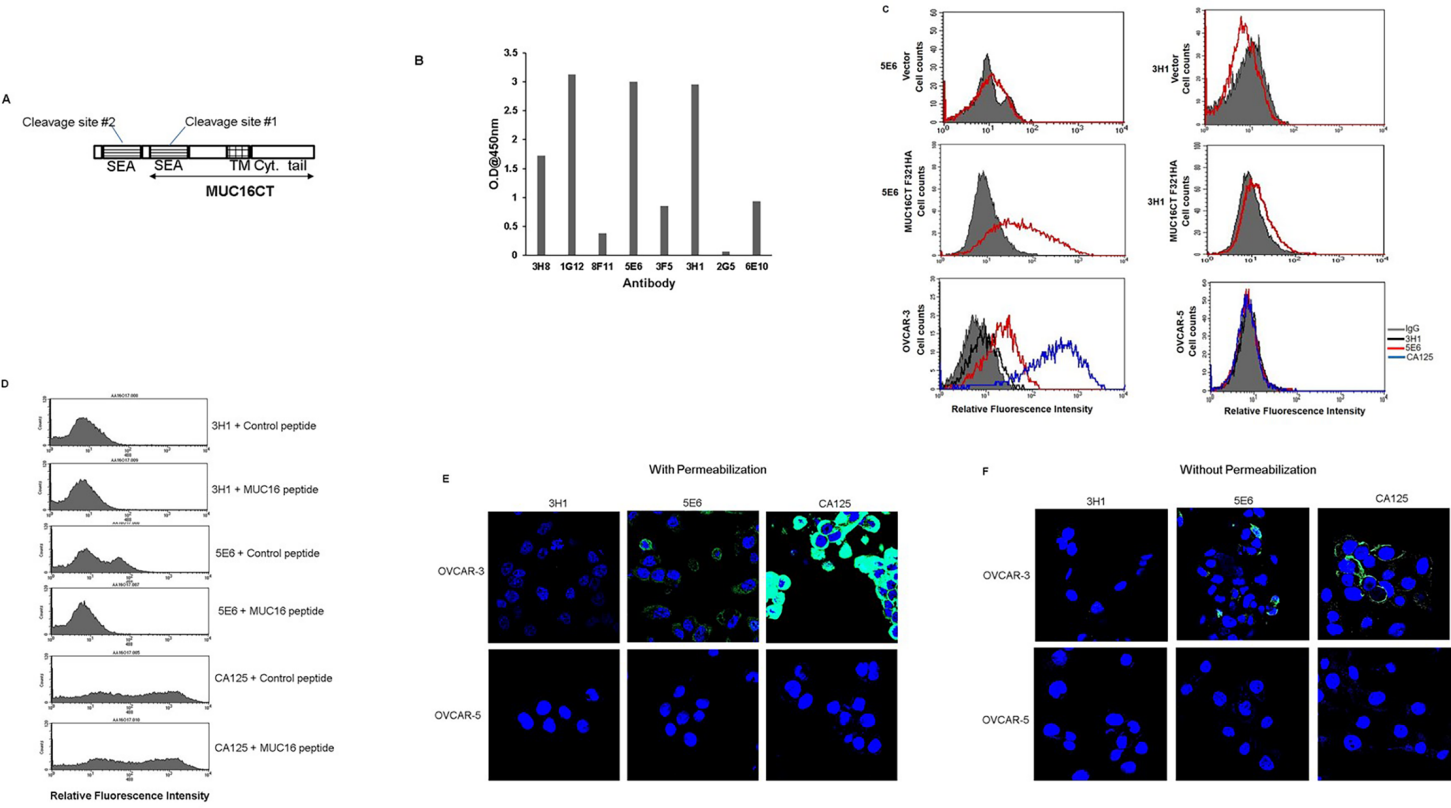
Generation and characterization of monoclonal antibodies (mAbs) to MUC16 C-terminal (CT) domain. (**A**) Structure of MUC16 CT domain indicating the two membrane-proximal cleavage sites. The fragment used for generation of hybridomas is indicated by a line with double arrow heads. This fragment incorporates the last putative cleavage site. Other important domains are indicated: TM- Transmembrane domain; Cyt. Tail- Cytoplasmic tail. (**B**) Binding of selected anti-MUC16 CT monoclonal antibodies with purified MUC16 CT protein using an indirect ELISA. MUC16 CT protein was coated in ELISA wells and hybridoma supernatants of the indicated antibodies were added. Antibody binding was detected using a secondary antibody labeled with horseradish peroxidase and TMB substrate. (**C**) Flow cytometry analysis showing relative binding of anti-MUC16 CT mAbs (5E6 and 3H1) to MIA PaCa-2 cells transfected either with control vector or MUC16 CT FL 321 construct (last 321 amino acids of MUC16). Binding was also analyzed on OVCAR-3 (MUC16_HIGH_) and OVCAR-5 (MUC16_LOW_) cells. Anti- tandem repeat mAb M11 served as a positive control. Cells were stained with the indicated antibodies and the signal was detected using Alexa-Fluor 488 anti-mouse IgG secondary antibody. A mouse IgG1 antibody served as the irrelevant isotype control and is indicated by the gray shaded curve. (**D**) Flow cytometry analysis of OVCAR-3 cells using mAbs pre-incubated with either MUC16 peptide or irrelevant control peptide. (**E and F**) OVCAR-3 and OVCAR-5 cells were seeded on coverslips, fixed with 4% Paraformaldehyde in PBS and were either permeabilized with 0.1% Triton X-100 in PBS (E) or not permeabilized (F) and incubated with 10 μg/ml of indicated mAbs. Signal was detected using Alexa Fluor 488 conjugated secondary antibody. Coverslips were placed on glass slides containing a drop of anti-fade Vectashield mounting medium and observed under a ZEISS confocal laser scanning microscope (magnification, 630X).

MUC16 has 56 SEA domains each of which has the potential to be cleaved. While it is predicted that the last and penultimate SEA domains could harbor the sites of cleavage [4, 13], recently using a series of constructs and transfection studies we demonstrated that MUC16 indeed undergoes cleavage, but the cleavage might not be happening in either of the last two proposed cleavage sites [13]. However, the endogenous existence of the MUC16 CT remains elusive. Therefore, it was highly desirable to obtain mAbs showing specificity to the retained membrane-anchored CT fragment. In order to develop antibodies, mice were immunized with last 114 amino acids of MUC16 C-terminus. This fragment incorporates part of the last SEA site but excludes the second to last or penultimate cleavage site (**Fig 1A**). Hybridoma supernatants were then screened using indirect ELISA (**Fig 1B**) assay and flow cytometry with MUC16 CT F321HA (last 321 amino acids of MUC16 C terminus) transfected MIA PaCa-2 cells (**Fig 1C**). Two reactive IgG1 clones were finally obtained that showed consistent reactivity–(3H1) and (5E6). A list of all the antibodies used in this study is given in **Table 1**. MAb 5E6 stained the surface of transfected cells better than mAb 3H1. The specificity of the mAbs was further tested for their ability to bind to the surface of ovarian cancer cells by flow cytometry (**Fig 1C and 1D**) and confocal microscopy (**Fig 1E and 1F**). For comparison of binding strength, the CA125 mAb (M11) (recognizing an epitope in the tandem repeat sequence of MUC16) was also included in the analyses. The mAb 5E6 stained the surface of OVCAR-3 cells, but as expected gave lower signal as compared to mAb M11. The CT antibodies (5E6, 3H1) and anti-tandem repeat antibody (M11) did not exhibit binding on OVCAR-5 cells, another ovarian cancer cell line that expresses low levels of MUC16 [22]. Pre-incubation with the immunizing MUC16 peptide abrogated the binding of mAb 5E6 but had no effect on the binding of M11 (**Fig 1D**). In immunofluorescence analysis with live cells, both mAb 5E6 and mAb M11 showed specific membranous staining while no staining was seen with mAb 3H1 and an appropriate isotype control (**S1 Fig**). In confocal analysis with fixed cells, mAb 5E6 predominantly stained cytoplasmic compartment while mAb M11 predominantly exhibited membranous staining. The mAb 3H1 failed to stain paraformaldehyde-fixed cells (**Fig 1E and 1F**).

**Table 1:** List of antibodies used in this study.

(Clone ID)	Immunogen	Epitope (location)	Isotype	Reference
(5E6)	Carboxy terminus (last 114 amino acids)	Juxtamembrane (extracellular)	IgG1(κ)	This study
(3H1)	Carboxy terminus (last 114 amino acids)	Juxtamembrane (extracellular)	IgG1(κ)	This study
LUM16-4 antiserum	Cytoplasmic tail peptide	Cytoplasmic tail (intracellular)	NA^a^	[16]
CA125 (M11)	CA125 antigen from ascites fluid	Tandem repeat (Extracellular)	IgG1	Dako, [21]
CA125 (OC125)	Ovarian mucin antigen	Tandem repeat (Extracellular)	IgG1	Fitzgerald Industries International

Nomenclature, isotype, origin and domain specificity of antibodies used in this study.

^a^Not applicable.

**Fig 2:**
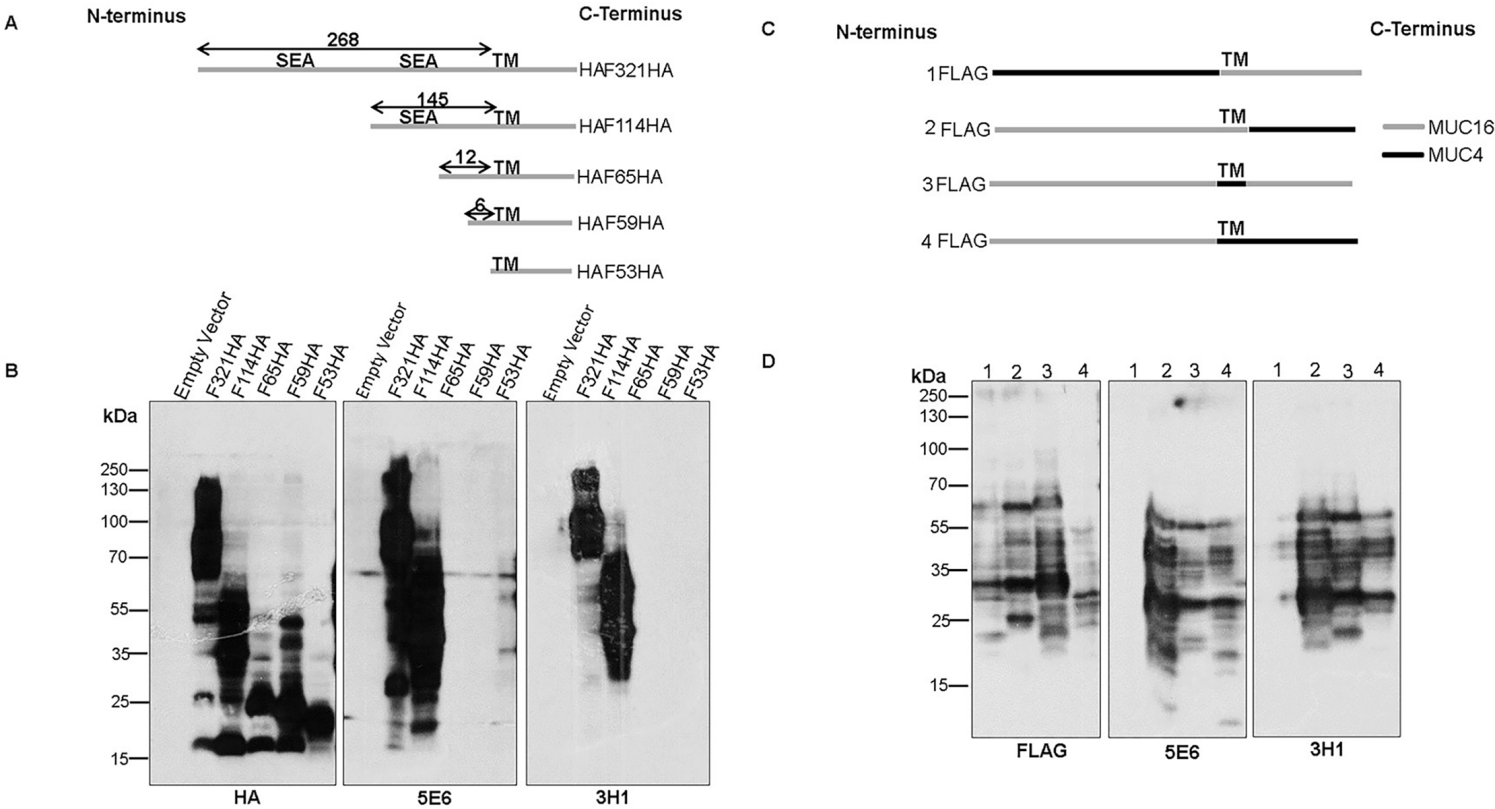
Partial epitope mapping using MUC16 CT constructs transfected into HEK293T cells. **(A**) Schematic representation of different lengths of MUC16 CT fragments with C-terminal HA-tag cloned into the p3X-FLAG-CMV9 vector (Empty vector) with a preprotrypsin leader peptide (LP). The predicted cleavage sites in the last (site #1, PLARRVDR) and penultimate (site #2, DSVLV) SEA domains and the transmembrane (TM) domain are indicated. (**B**) Partial epitope mapping using various constructs of MUC16 CT (given in (**A**)) transfected into HEK293T cells was performed. Lysates from transfected cells were immunoblotted with the indicated antibodies. (**C**) The Flag tagged F114HA MUC16 CT construct was domain swapped with the various domains of MUC4 as indicated. (**D**) The constructs described in (**C**) were transfected into HEK293T cells. Lysates from these cells were immunoblotted with the respective antibodies.

**Fig 3:**
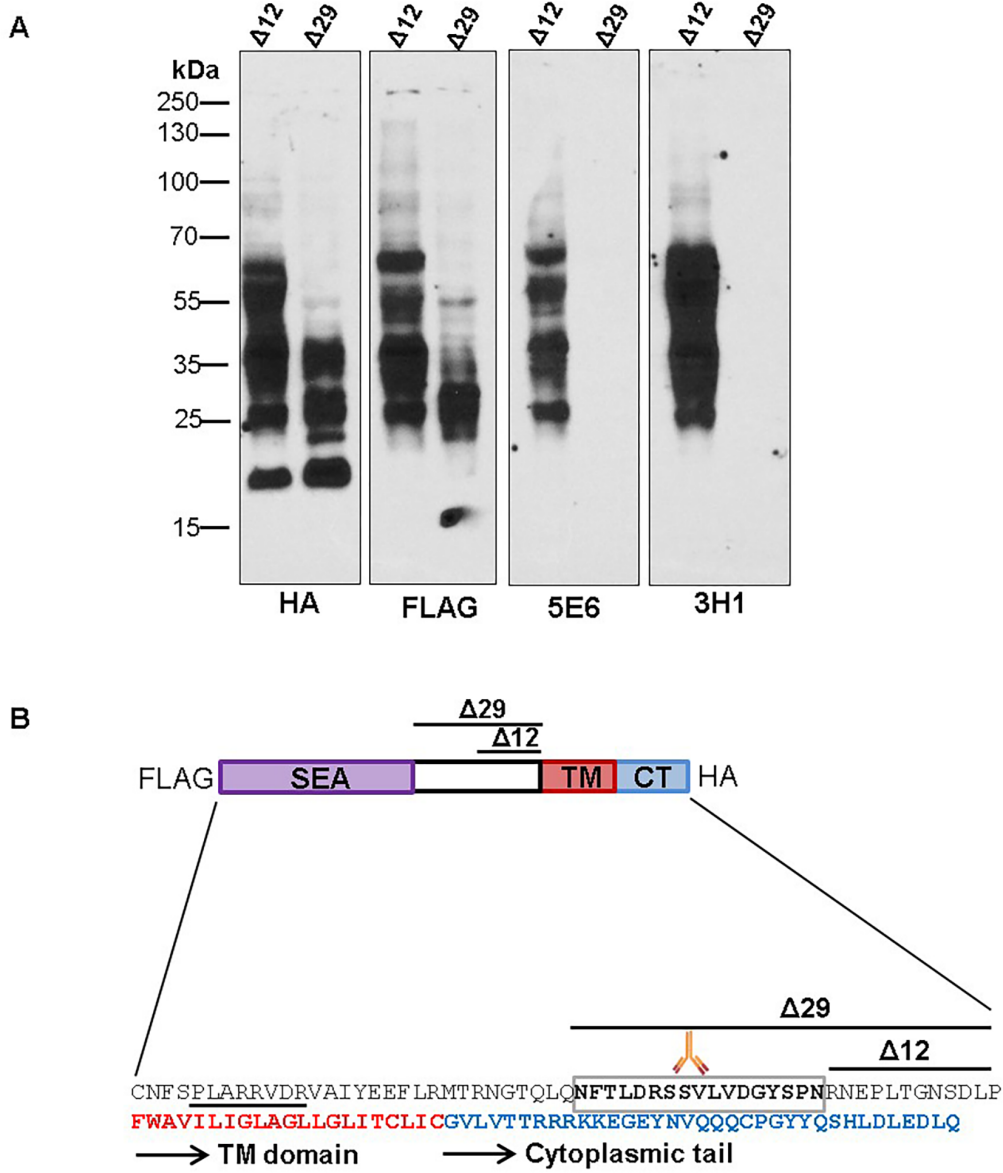
Narrowing down the epitope recognized by MUC16 CT mAbs. (**A**) The last 12 and 29 amino acids from TM domain of MUC16 were deleted in the F114HA construct, tagged with FLAG at N-terminus and HA at C-terminus, to generate 2 deletion constructs (Δ12 and Δ29). The Δ12 and Δ29 constructs were transfected into HEK293T cells and immunoblotted with the indicated antibodies. (**B**) Schematic representation of MUC16 C-terminal region indicating various domains, cleavage site and approximate location of the putative epitope recognized by mAbs 5E6and 3H1. Key: Purple—represents the last SEA domain. Red portion—indicates the start of the transmembrane domain. Boxed region—indicates the putative epitope. Underlined region—represents the predicted putative cleavage site.

To determine the epitopes recognized by the new antibodies, HEK293T cells were transfected with constructs encoding MUC16 CT fragments of varying lengths (**Fig 2A**) and the cell lysates were analyzed by western blot assays as described previously [13]. Both CT mAbs recognized the 321 and 114 amino acid MUC16 C-terminus constructs but could not recognize constructs representing the last 65, 59 and 53 amino acids (**Fig 2B**). To examine the possible contribution of cytoplasmic, transmembrane, or extracellular sequences in the reactivity of mAbs 3H1 and 5E6, the corresponding MUC16 regions in the MUC16 CT F114HA construct were exchanged with those of another transmembrane mucin, MUC4 **(Fig 2C**). Both mAbs tolerated changes in the cytoplasmic and transmembrane domain but could not tolerate changes in the extracellular sequences thus suggesting that their reactivity is directed towards extracellular epitope(s) (**Fig 2D**). Finally, 12 or 29 amino acids (**Δ12 and Δ29**) lying N-terminal to the transmembrane side were deleted from the MUC16 CT F114HA construct and the newly formed constructs were transfected into HEK293T cells. Both antibodies exhibited reactivity with the protein encoded by Δ12 construct but failed to recognize Δ29 product (**Fig 3A**), suggesting that these antibodies recognize a 17 amino acid region that lies 12 amino acids N-terminal to the transmembrane domain (**Fig 3B**). Interestingly the site lies C-terminal to the last proposed SEA cleavage site.

### MUC16 is cleaved and the resulting fragments remain associated with each other

**Fig 4:**
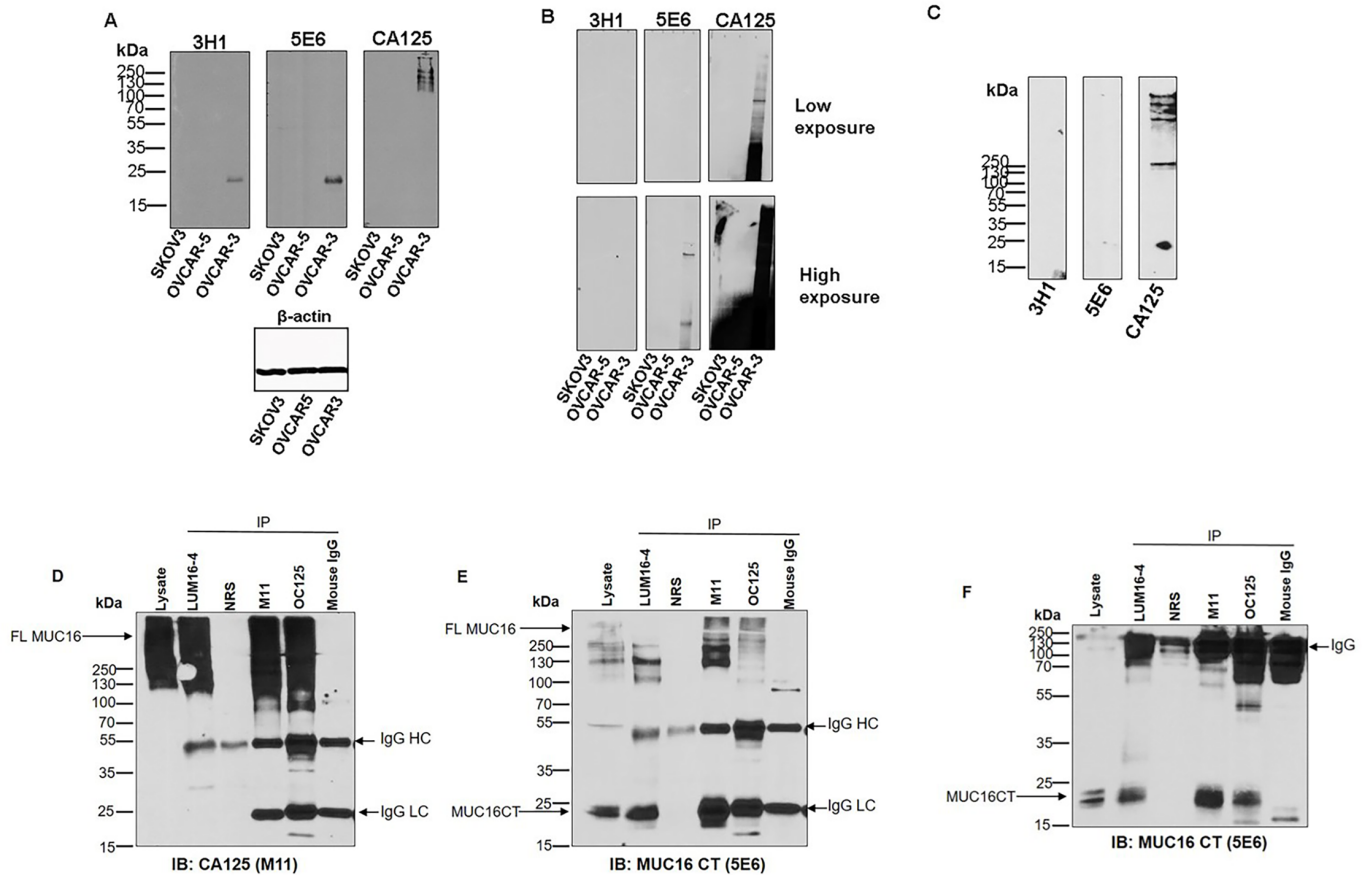
MUC16 is cleaved and a fraction of cleaved fragments remain non-covalently associated in the cells. Lysates of ovarian cancer cell lines (SKOV3, OVCAR-5 and OVCAR-3) were resolved on 12% SDS-PAGE (**A**) or 0.1% SDS-2% agarose gels (**B**), transferred to PVDF membranes and probed with the indicated antibodies. (**C**) OVCAR-3 culture supernatant was resolved on a gradient (4–12%) SDS-PAGE, transferred to PVDF membranes and probed with the indicated antibodies. (**D, E and F**) OVCAR-3 cells were lysed and precipitated with MUC16 CT antiserum (LUM16-4) or CA125 mAbs (M11 and OC125) as described in the Materials and Methods section. The precipitates were resolved by SDS-PAGE under reducing (**D and E**) and non-reducing conditions (**F**) followed by transfer to PVDF membrane and probed with the indicated antibodies. Mouse IgG1 and non-specific rabbit serum (NRS) were used as negative controls. Full length MUC16 (FL MUC16) was detected as a high molecular weight smear recognized by both CA125 and 5E6 mAbs. MAb 5E6 recognized the cleaved C-terminal fragment of MUC16 (MUC16 CT). Intact non-reduced immunoglobulin (IgG) and reduced heavy and light chains (IgG HC and IgG LC respectively) are indicated.

**Fig 5:**
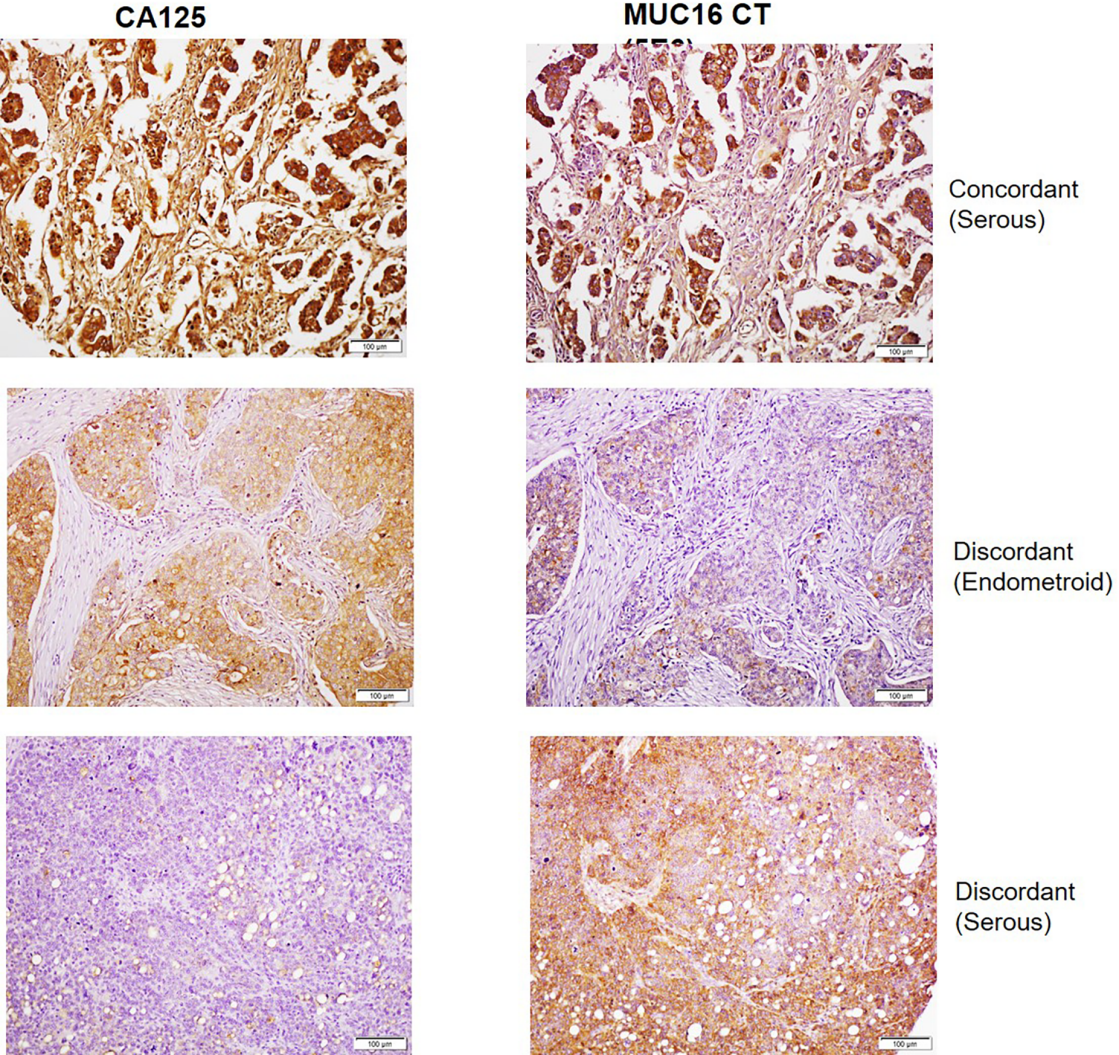
Comparative analysis of CA125 and MUC16 CT staining in human ovarian cancer cases by IHC. Duplicate ovarian cancer tissue microarrays (OV1004, BIOMAX) were processed for IHC staining as described in the Materials and Methods section and with primary antibodies CA125 (M11) and MUC16 CT (5E6). Representative cases of concordant and discordant staining are indicated. The top and bottom panels represent serous subtypes which exhibited intense staining with both CA125 and MUC16 CT mAbs. The middle panel represents the endometroid subtype that exhibited positive reactivity with CA125 but demonstrated comparatively lower staining with MUC16 CT mAb.

To determine the existence of anti-MUC16 CT mAb reactive proteolytic cleavage products originating from native MUC16 backbone, lysates of ovarian cancer cell lines expressing high levels of endogenous MUC16 were analyzed by western blotting. In SDS-PAGE based western blots, both mAbs 5E6 and 3H1 identified a 20 kDa band in the lysates of MUC16 expressing OVCAR-3 cells but not in SKOV3 and OVCAR-5 cells which express low levels of MUC16 (**Fig 4A**) while the anti-MUC16 tandem repeat mAb M11 (CA125) showed reactivity in OVCAR-3 lysate as a high molecular weight smear. Upon higher exposure, mAb 5E6 also displayed a higher molecular weight smear similar to what is seen with mAb M11 (**S2 Fig**). In SDS-agarose gel-based western blotting, anti- tandem repeat mAb M11 (CA125) recognized a smear in OVCAR-3 lysate that is characteristic of MUC16, while anti-MUC16 CT mAb 5E6 exhibited reactivity to distinct high molecular weight bands corresponding to the dominant bands recognized by mAb M11.(**Fig 4B**). However, mAb 3H1 did not recognize MUC16 in SDS-agarose immunoblotting. Analysis of the enriched culture supernatant of OVCAR-3 cells indicated the presence of M11 reactive bands that are possibly cleaved from the cell surface. However, the anti-CT mAbs failed to recognize the released MUC16 in the cell culture supernatant suggesting that the reactive CT epitopes are retained on the cell following cleavage (**Fig 4C**). To examine if the M11 reactive tandem repeat fragment and CT fragment can remain non-covalently associated following cleavage, detergent extracted lysate from OVCAR-3 cells was immunoprecipitated using CT antiserum LUM16-4 and CA125 antibodies (M11 and OC125 type) and subsequently probed with mAb M11 or 5E6 (**Fig 4D, 4E and 4F**). Both CA125 mAbs (M11 and OC125) and LUM 16–4 immunoprecipitated full-length MUC16 that was detected when the immune complexes were probed with mAbs CA125 (M11) (**Fig 4D**) and 5E6 (**Fig 4E and 4F**). In addition to recognizing full-length MUC16, anti- MUC16 CT mAb 5E6 recognized the cleaved 20 kDa band in the immune-complexes immuoprecipitated with both CT antiserum (LUM 16–4) and CA125 mAbs (M11 and OC125) (**Fig 4E and 4F**). Since the LUM16-4 antiserum was raised against a cytoplasmic tail peptide sequence, this further shows that the 20 kDa band represents the cleaved C-terminal fragment of MU16. Together these observations suggest that most MUC16 remains intact in the cells (suggested by the recognition of high molecular bands by CA125 and CT mAbs) while a fraction of MUC16 is cleaved. The fraction of cleaved CA125-type fragment can be released from the cells (based on positive reactivity of M11 in culture supernatants), while a fraction of cleaved fragments remain non-covalently associated on the cells and can be immunoprecipitated by both CA125 mAbs and cytoplasmic tail specific LUM16-4 antiserum.

### Comparative analysis of CA125 and MUC16 CT mAbs on ovarian cancer tissues by immunohistochemistry (IHC)

**Fig 6:**
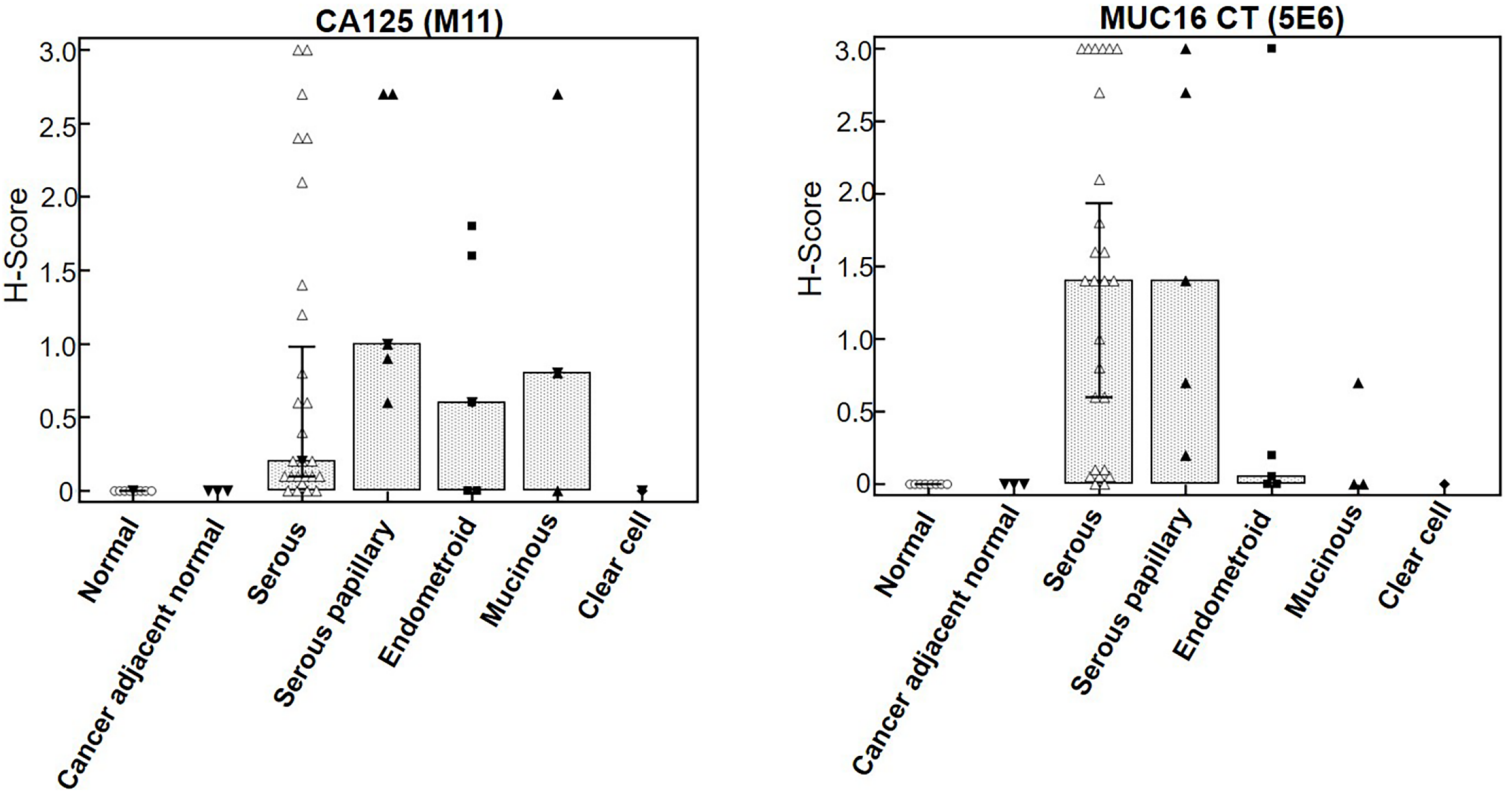
Box plot comparing CA125 and MUC16 CT staining across various histologic types of ovarian cancer. MUC16 immunoreactivity was higher in the serous and serous papillary adenocarcinoma tissues as compared to other types. The CA125 mAb also exhibited reactivity with endometroid and mucinous types while CT mAb failed to detect these types efficiently. No staining is observed in normal or cancer adjacent normal ovarian tissue with either CA125 or MUC16 CT mAbs.

**Fig 7:**
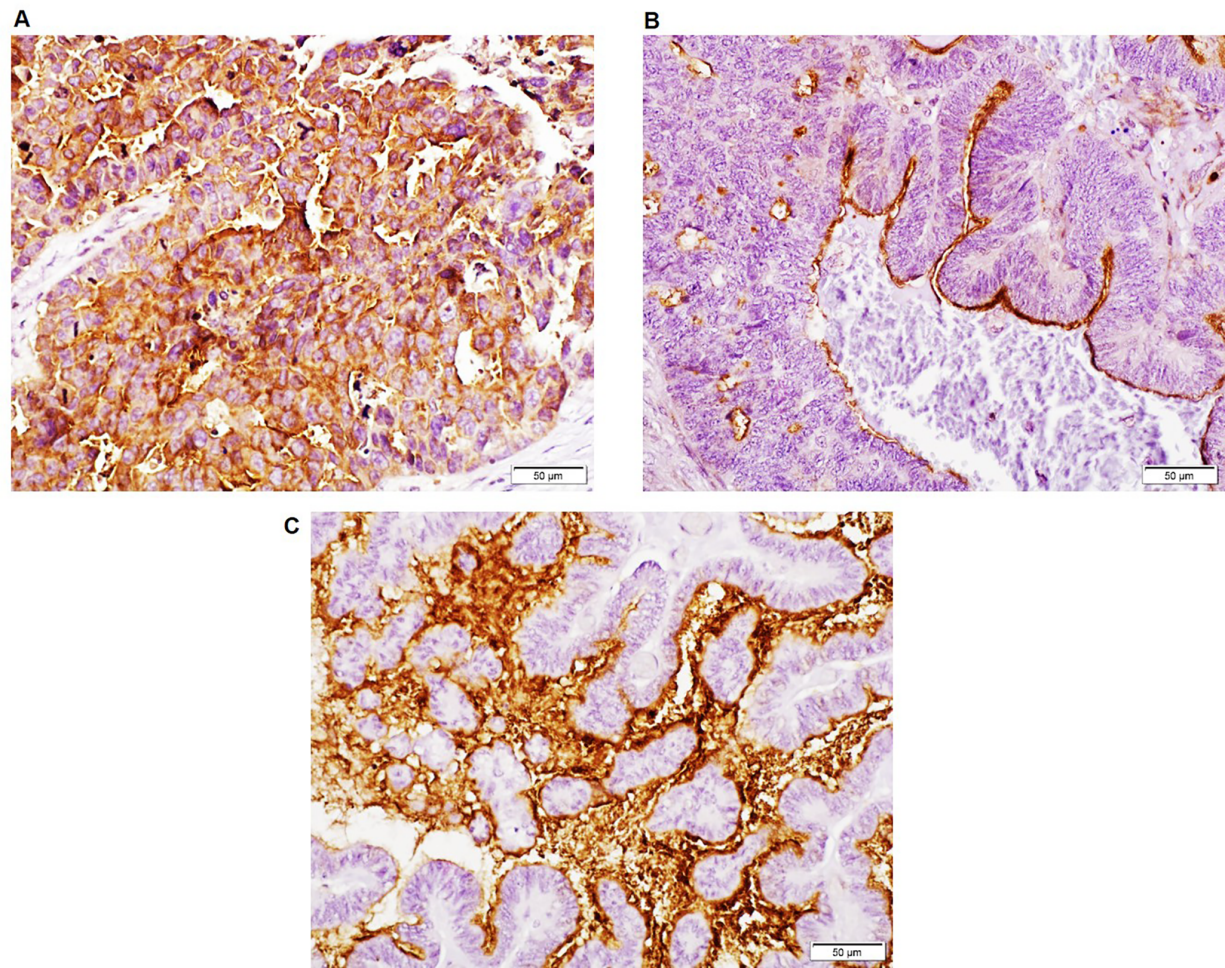
MUC16 CT mAb 5E6 exhibits heterogeneous staining on human ovarian cancer tissues. Sections of ovarian cancer tissues indicating strong membranous and cytoplasmic staining in tumor cells (A), focal apical membranous staining on tumor cells (B), and strong intra-luminal and membranous staining (C) of MUC16 CT. In all cases, the surrounding stroma was negative for MUC16 CT expression. Original magnification 200х.

Initial optimization of CT mAbs for IHC was performed on selected ovarian cancer tissue sections using mAb M11 as positive control. Focal staining was observed with mAb 3H1 in ovarian cancer tissue sections, while mAb 5E6 exhibited a more extensive reactivity pattern that was reflective of CA125 staining. Therefore, the reactivity of mAb 5E6 was compared with CA125 on a tissue microarray comprising sections from normal and cancer adjacent normal ovarian tissues (n  =  9), and tissues from primary ovarian cancer (OC) of various grades (n  =  40) (**Fig 5**). The 5E6 antibody exhibited positive staining in 33/40 (82.5%) cases while CA125 stained 32/40 (80%) cases of ovarian cancer (**Fig 6**). Both mAbs showed no staining in normal ovarian or cancer adjacent normal ovarian tissue (0/9). MAb 5E6 stained 29/31 (93.5%) cases of serous and serous papillary adenocarcinoma while CA125 stained 27/31 (87%) cases. However, mAb 5E6 performed poorly in recognizing cases other than serous and serous papillary subtypes, as compared to CA125 (**Fig 6**), possibly due to the lack of epitope multiplicity. Out of 5 endometroid cases, mAb 5E6 stained only 1 case strongly and exhibited weak reactivity in 2 cases, while CA125 mAb exhibited strong reactivity with 3 specimens. Out of 3 cases of mucinous adenocarcinoma mAb 5E6 stained 1 case weakly while CA125 mAb exhibited moderate staining in 2 cases. Of the total 33 positive cases, mAb 5E6 exhibited a more widespread staining of cancer cells in 25 cases (percentage area stained >70%) whereas in 8 specimens it exhibited a focal staining. MAb CA125 exhibited widespread staining in 20 specimens out of total of 32 positives cases while 12 cases showed focal staining. A high concordance was observed in the positive cases between CA125 and MUC16 CT (37/49 (75%) cases), but some discordant staining was also noted (12/49 (24%) cases) (**Fig 5**). Out of 12 discordant cases, in 8 cases the mAb 5E6 exhibited higher staining while in remaining 4 cases, CA125 mAb staining was better. MAb CA125 staining was mostly membranous and cytoplasmic while mAb 5E6 staining was mostly cytoplasmic even though strong membrane staining could be seen in some cases. The mAb 5E6 staining was also highly heterogeneous, varying not only from case to case but also within the same section. On average cytoplasmic staining was common (**Fig 7A**); but localized apical membranous staining was also observed in some cases (**Fig 7B and 7C**). No correlation was observed between CT mAb staining and tumor grade, stage or type except the fact that CT mAb predominantly stained serous and serous papillary as compared to endometroid, mucinous and clear cell carcinoma cases (**Fig 6**).

## Discussion

The presence of MUC16 CT has never before been biochemically described in ovarian cancer. OVCAR-3 cells are ideally suited for dissecting the function of MUC16 since these cells express comparable amounts of surface expressed MUC16 as seen in ovarian cancer patients, maintain an epithelial phenotype and have been extensively used in several studies involving MUC16 [9, 17, 23, 24]. Novel antibodies were raised against the juxtamembrane region of MUC16 that recognized the OVCAR-3 cell surface in flow cytometry. Despite having apparent specificity to the same site, mAb 5E6 exhibit stronger biding to the cell surface than the mAb 3H1 possibly due to a more favorable conformation of 5E6 epitope. Alternatively, it is also likely the mAb 3H1 reactive epitope is either inaccessible or disrupted by cleavage. Although multiple cleavage sites exist in MUC16 backbone, our observations on the surface retention of MUC16 are limited to the epitope recognized by our mAbs. Development of additional mAbs to other regions of MUC16 CT sequence could provide a clearer picture.

Human MUC16 is known to be expressed on the cell surface. It has also been demonstrated that the C-terminal domain of MUC16 that lacks a signal sequence has the capability to be routed via the traditional ER-Golgi secretory pathway to the cell surface [25]. Our recent studies using MUC16 CT transfected HeLa cells, indicated cleavage occurs in the acidic Golgi compartments [13]. Using MUC16 CT transfected cells we and others have shown that the MUC16 CT confers tumorigenic, metastatic, and anti-apoptotic, properties to cancer cells and contribute to therapy resistance [5, 7, 8, 12, 14], however the underlying mechanisms remain poorly understood. The new anti MUC16 CT mAbs can serve as useful tools for elucidating the mechanisms by which MUC16 contributes to pathobiology of ovarian and other MUC16-overexpressing malignancies.

Using a series of deletion constructs of MUC16, the epitope for both CT mAbs was mapped to a 17 amino acid region 12 amino acid outside of the identified transmembrane domain. Domain swapping of the intracellular or transmembrane regions had no effect on the reactivity of both the antibodies in immunoblot analysis suggesting that the antigenic determinant was extracellular. Both CT mAbs recognized 20kDa band in the lysate of OVCAR-3 cells. This band contains the cytoplasmic tail portion of MUC16 since it was immunoprecipitated by LUM16-4 antiserum. Intriguingly, both anti-MUC16 tandem repeat mAbs (M11 and OC125) also immunoprecipitated MUC16-CT mAb reactive fragments providing unequivocal evidence that the cleaved MUC16 fragments remain associated. Analysis of cell lysates by immunoblotting and immunoprecipitation and enriched culture supernatant by immunoblotting further revealed the existence of MUC16 in both intact and cleaved forms. While most MUC16 remains intact in the cells as suggested by the recognition of high molecular bands by CA125 and CT mAbs (**Fig 4A and 4B**), a fraction of MUC16 is cleaved. The fraction of cleaved CA125-type fragment can be released from the cells (based on positive reactivity of M11 in culture supernatants-**Fig 4C**), while a fraction of cleaved fragments remains non-covalently associated on the cells and can be immunoprecipitated by both CA125 mAbs and LUM16-4 antiserum raised against MUC16 cytoplasmic tail (**Fig 4D, 4E and 4F**).

Unlike LUM16-4 anti-serum, both MUC16 CT mAbs were unable to immunoprecipitate CA125-CT complex from OVCAR-3 lysate (**S3 Fig**) possibly due to steric hindrance caused by the glycosylated larger CA125-type fragment. However, one of the CT mAbs, 5E6, was able to bind to the OVCAR-3 cell surface. It is possible that increased CA125 shedding following cleavage exposes the 5E6 epitope and lead to enhanced cell surface binding. Our findings that MUC16 is cleaved and the cleaved fragments remain associated are similar to what has been observed for another transmembrane mucin MUC1. MUC1 undergoes cleavage to form a ~30kDa fragment (longer cytoplasmic tail) that remains associated with the N-terminal fragment as a heterodimeric complex that is then transported to the cell surface. The larger N-terminal subunit is then released from the cell surface due to reasons not completely understood [26–29]. Hence the original idea by Wreschner *et al*. [30] seems to hold true as we find yet another mucin with SEA domains that undergoes cleavage and the cleaved fragments remain associated. It will be of interest to investigate the underlying mechanisms of cleavage and its biological consequences. Although our recent studies have demonstrated that MUC16 cleavage occurs in Golgi compartment under acidic pH, those studies were performed using overexpression of recombinant fragments. The new anti-MUC16 CT mAbs will serve as valuable reagents to elucidate the mechanisms and determine the functional significance of cleavage of native MUC16. These mAbs may help identify biological triggers and signaling events that induce the cleavage of MUC16 in cancer cells. Ovarian tumors exhibit strong positivity with CA125 mAbs but there is considerable variation in the observed reactivity depending on the mAb employed and histological subtypes. Mostly MUC16 is expressed in 56–85% of serous cases, while the incidence of CA125 positivity in endometroid, mucinous and clear cell types is relatively lower [31, 32]. A similar pattern was observed in the positivity rates of CT mAb 5E6 in the present study. CA125 was sensitive enough to detect cases of mucinous and endometroid due to the tandem repeat nature of the epitope. The CT mAb 5E6 showed minimal reactivity in these cases and was more specific to serous and serous papillary cases. The staining was predominantly membranous for CA125 while for 5E6 it was predominantly cytoplasmic. Interestingly, the overall detection rate of serous and serous papillary cases by CT mAb 5E6 was slightly higher than that observed with CA125 (93.5% as compared to 87%). The lower reactivity of CA125 in serous tissues where MUC16 CT mAb was positive might be due to extentsive O-glycosylation of CA125 epitopes or enhanced shedding and subsequent loss from cell surface. In contrast, the binding of the 5E6 mAb might not be affected by glycosylation and/or shedding and hence may serve as a more accurate MUC16 indicator in cancer cases. Indeed, the glycosylation-mediated masking effect and sequestration by circulating CA125 in the serum may be the primary reasons for the limited success of CA125 mAb-based therapies in clinical trials [33, 34]. The CT mAbs described in the present study may circumvent both of these problems and may hold promise as future candidates for development of targeted therapeutic agents. Surprisingly, mAb 5E6 exhibited intraluminal staining in some ovarian cancer cases suggesting that MUC16 CT can also be released from the cells (**Fig 7C**). However, these observations are in line with some studies that suggest that intact MUC16 can be secreted from the cells [16]. However, we could not detect MUC16 in OVCAR-3 culture supernatant when using 3H1 and 5E6 mAbs in western blotting (**Fig 4C**). As a final note, we observed focal reactivity of mAb 3H1 in few tumors samples. It is possible that the 3H1 antibody recognizes a specific conformation of the molecule or its epitope may be blocked by post translational modifications. It is possible that its reactivity might be indicative of particular conditions such as cleavage.

In conclusion, we have developed highly sensitive reagents to probe the functions of MUC16 CT that is retained on the cell surface after cleavage. One of the CT mAbs (5E6) demonstrates equivalent sensitivity in immunohistochemistry based detection of ovarian cancer cases as compared to tandem repeat epitope recognizing mAb (CA125). The proximity of 5E6 and 3H1 epitopes to the transmembrane domain allows these mAbs to bind to the cell surface that can be exploited for developing MUC16 targeted therapeutics for ovarian cancer.

## Supporting information

S1 Fig Immunofluorescence analysis on live OVCAR-3 cells. Immunofluorescence analysis on live OVCAR-3 cells showing cell surface staining for mAbs 5E6 and M11. Live cells were stained with the indicated antibodies and the signal was detected using Alexa-Fluor 488 anti-mouse IgG secondary antibody. The cell suspension was directly observed under EVOS FL Auto Cell Imaging System.
(TIF)

S2 Fig Overexposed blot of Fig 4A. Overexposed blot of Fig 4A showing the high molecular weight MUC16 recognized by mAb 5E6 similar to that of mAb CA125 but with lower intensity.
(TIF)

S3 Fig Immunoprecipitation assay determining the ability of mAbs to recognize CA125-CT complex in OVCAR-3 detergent extracted lysate. OVCAR-3 cells were lysed and immnoprecipitated with MUC16 CT mAbs 5E6 and 3H1 and CA125 mAb (M11) as described in the Materials and Methods section. The immune complexes were resolved by SDS-PAGE followed by transfer to PVDF membrane and probed with the indicated antibodies. Irrelevant mouse IgG1 was used as an isotype control. MAb 5E6 recognized the cleaved cytoplasmic tail of MUC16 (MUC16 CT) that is indicated by an arrow.
(TIF)

S1 File NC3Rs ARRIVE guidelines checklist. (PDF)
