# Supplementary figures and images for: Development and characterization of carboxy-terminus specific monoclonal antibodies for understanding MUC16 cleavage in human ovarian cancer
Source: PLoS One. 2018 Apr 30;13(4):e0193907. doi: 10.1371/journal.pone.0193907 (PMC5927449; doi:10.1371/journal.pone.0193907)

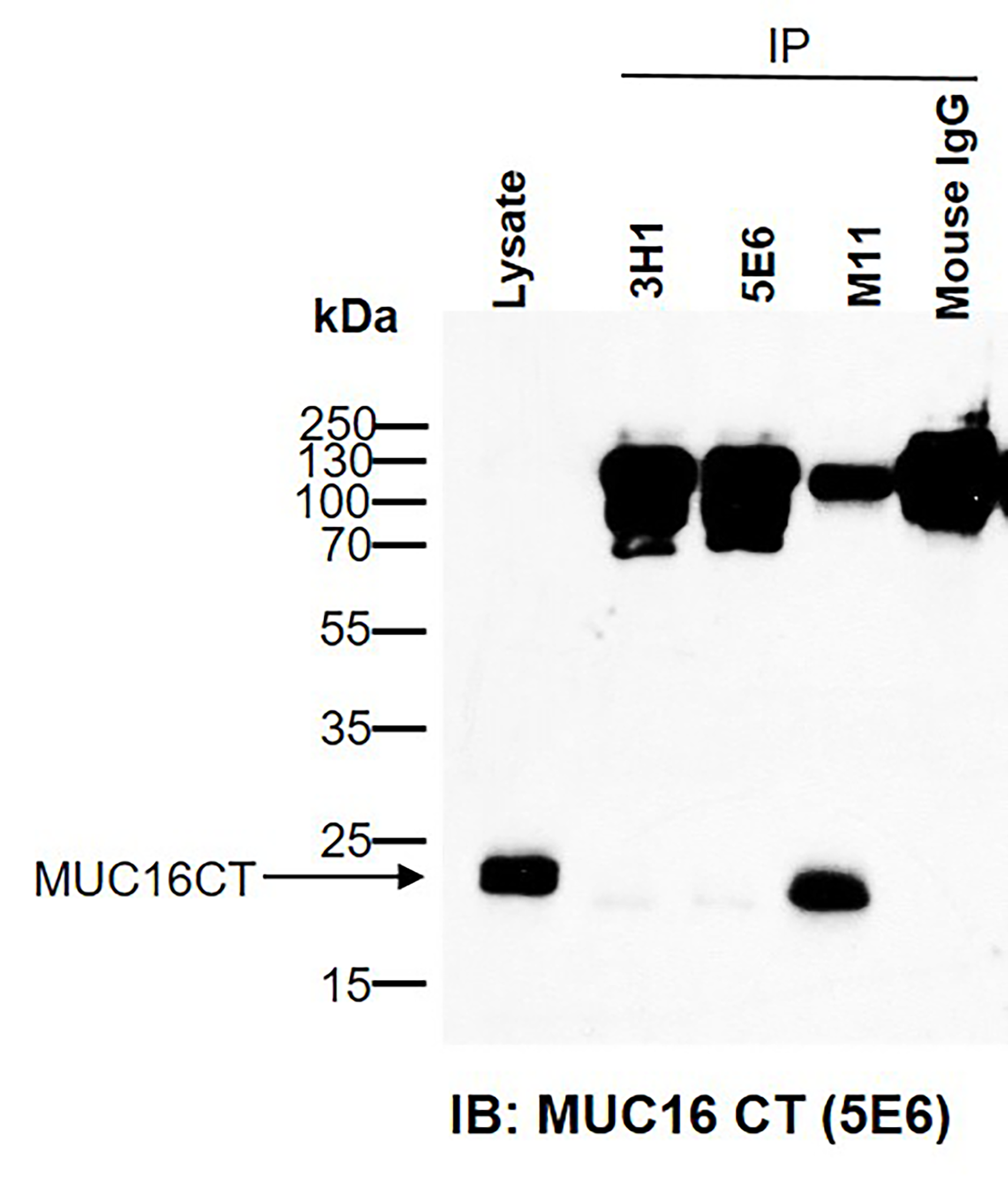

Supplement: S1 Fig — Immunofluorescence analysis on live OVCAR-3 cells showing cell surface staining for mAbs 5E6 and M11. Live cells were stained with the indicated antibodies and the signal was detected using Alexa-Fluor 488 anti-mouse IgG secondary antibody. The cell suspension was directly observed under EVOS FL Auto Cell Imaging System. (TIF) [file pone.0193907.s001.tif]

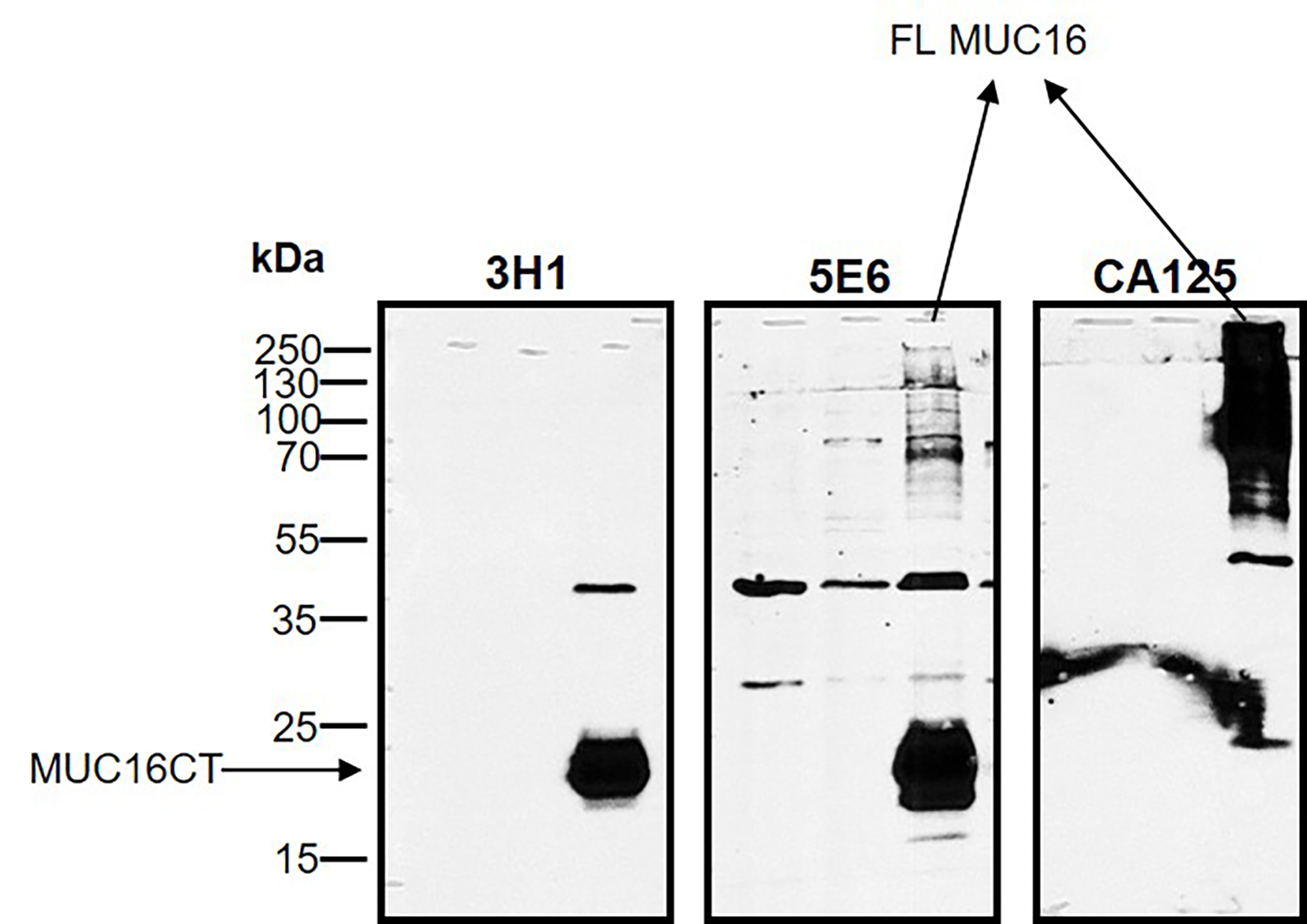

Supplement: S2 Fig — Overexposed blot of Fig 4A showing the high molecular weight MUC16 recognized by mAb 5E6 similar to that of mAb CA125 but with lower intensity. (TIF) [file pone.0193907.s002.tif]

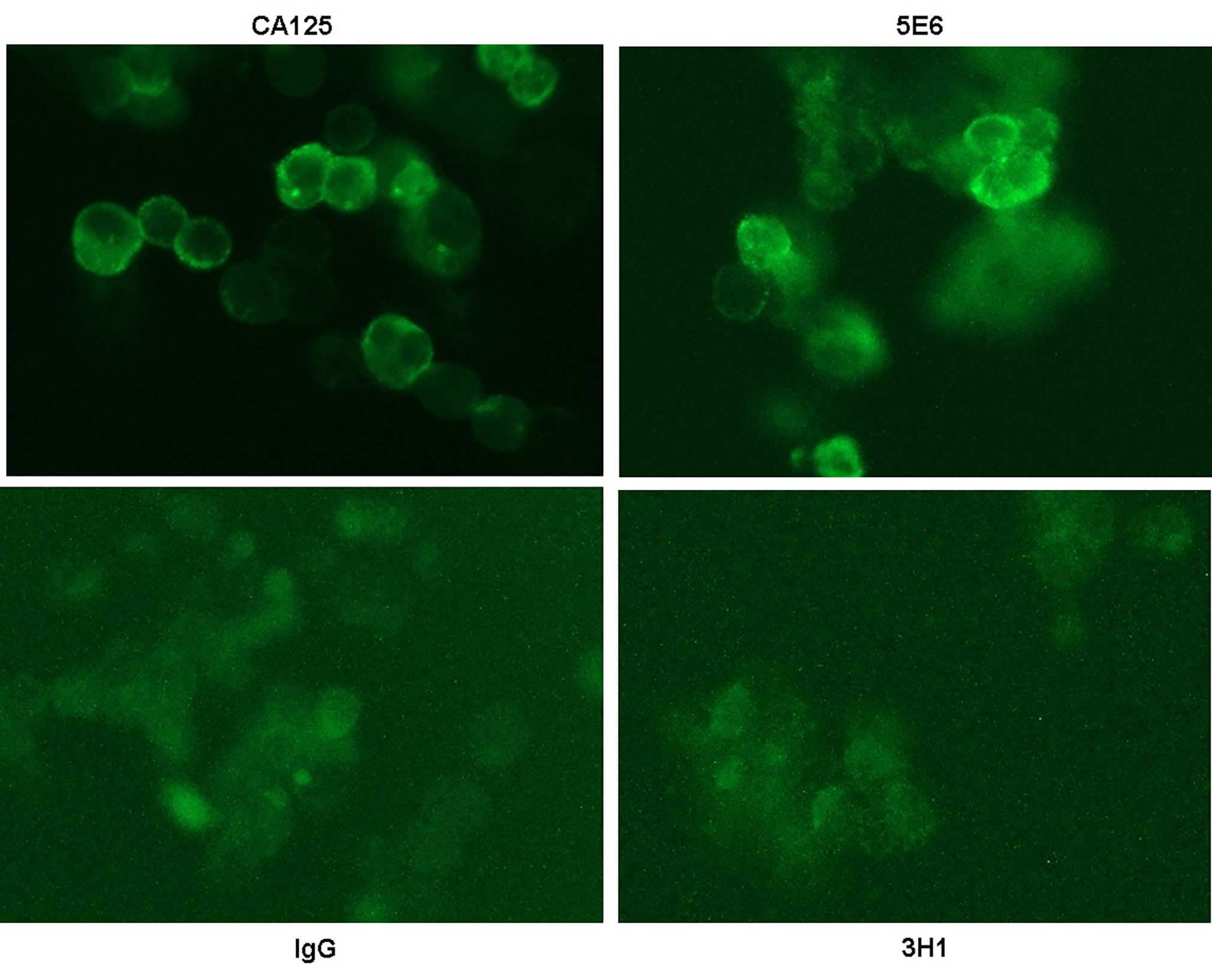

Supplement: S3 Fig — OVCAR-3 cells were lysed and immnoprecipitated with MUC16 CT mAbs 5E6 and 3H1 and CA125 mAb (M11) as described in the Materials and Methods section. The immune complexes were resolved by SDS-PAGE followed by transfer to PVDF membrane and probed with the indicated antibodies. Irrelevant mouse IgG1 was used as an isotype control. MAb 5E6 recognized the cleaved cytoplasmic tail of MUC16 (MUC16 CT) that is indicated by an arrow. (TIF) [file pone.0193907.s003.tif]
